# Search for Dislocation Free Helium 4 Crystals

**DOI:** 10.1007/s10909-014-1251-0

**Published:** 2014-12-02

**Authors:** F. Souris, A. D. Fefferman, A. Haziot, N. Garroum, J. R. Beamish, S. Balibar

**Affiliations:** 1 Laboratoire de Physique Statistique de l’École Normale Supérieure associé au CNRS et aux Universités P.M. Curie et D. Diderot, 24 rue Lhomond, 75231 Paris Cedex 05, France; 2 Present Address: CNRS Institut NÉEL, Université Grenoble Alpes, BP 166, 38042 Grenoble Cedex 9, France; 3 Present Address: Department of Physics, University of Alberta, Edmonton, AB T6G 2E1 Canada; 4 Present Address: Department of Physics, Pennsylvania State University, University Park, PA 16802 USA

**Keywords:** Solid helium, Dislocations, Plasticity

## Abstract

The giant plasticity of $^4$He crystals has been explained as a consequence of the large mobility of their dislocations. Thus, the mechanical properties of dislocation free crystals should be quite different from those of usual ones. In 1996–1998, Ruutu et al. published crystal growth studies showing that, in their helium 4 crystals, the density of screw dislocations along the c-axis was less than 100 per cm$^2$, sometimes zero. We have grown helium 4 crystals using similar growth speeds and temperatures, and extracted their dislocation density from their mechanical properties. We found dislocation densities that are in the range of 10$^4$–10$^6$ per cm$^2$, that is several orders of magnitude larger than Ruutu et al. Our tentative interpretation of this apparent contradiction is that the two types of measurements are somewhat indirect and concern different types of dislocations. As for the dislocation nucleation mechanism, it remains to be understood.

## Introduction

Crystals with free moving dislocations are known to be softer than when the dislocation motion is damped or pinned by some mechanism [1, 2]. Recent studies of $^4$He crystals in Edmonton and in Paris [3–7] have illustrated this general property. In the absence of binding of $^3$He impurities to dislocations and at temperatures low enough for dislocations to move with negligible collisions with thermal phonons, a very large softening called “giant plasticity” occurs in $^4$He crystals. It can be understood as a consequence of the free gliding of dislocations between network nodes, where dislocations are strongly pinned. They glide parallel to the basal planes of the hexagonal structure so that the elastic coefficient $c_{44}$ nearly vanishes. Our recent studies show that dislocation lines behave as elastic strings as if the periodic lattice did not introduce any measurable “Peierls energy barriers” against their motion.

When fitting their data with the theory of Granato and Lücke [2] and its development by Ninomiya [8], Haziot et al. [5] could extract the density of dislocations $\Lambda$ in their crystals and the length between nodes in their network, that is their “network length” $L_N$. They found dislocation densities in the range of 10$^4$–10$^6$ cm$^{-2}$ and network lengths from 50 to 200 $\mathrm{\mu }$m depending on growth conditions. Given the value of $\Lambda$, these lengths are much longer than would be expected if dislocation lines formed an isotropic homogeneous 3D network. They must be aligned parallel to each other so that they avoid crossings. This result is consistent with X-ray images by Iwasa et al. [9] who found that dislocations are aligned parallel to each other with Burgers vectors parallel to the basal planes, and in sub-boundaries that are perpendicular to the basal planes. Later on, Fefferman et al. [7] showed that the dislocation network is far from perfectly ordered so that there is a rather large distribution of network lengths in the crystals they studied, typically from tens to hundreds of microns so that the length $L_N$ determined by Haziot et al. [5] is some kind of mean value of this distribution.

In 1996–1998, Ruutu et al. [10, 11] demonstrated the possibility to grow crystals free of screw dislocations along the c-axis. Crystals completely free of dislocations should behave quite differently from those with dislocations. They should have no elastic anomaly, in contrast to crystals with even small dislocation densities, which should have anomalous elastic properties because dislocations could be as long as several millimeters. They would then resonate in the kHz frequency range used by the Edmonton and Paris groups for stiffness measurements. This has been our motivation to grow crystals as carefully as possible in an acoustic cell where their stiffness could be measured at low frequency. We used growth rates and growth temperatures similar to those shown by Ruutu et al. to lead to excellent crystals in 1996–1998. Ruutu et al. studied the growth dynamics of faceted $^4$He crystals grown very slowly (less than 200 nm/s) below 20 mK. They identified regimes typical of the classical “spiral growth” due to low densities of screw dislocations emerging at the surface of facets. From the observed pressure threshold below which no growth took place, they deduced densities of screw dislocations along the c-axis, which were less than $100$ cm$^{-2}$, a very low value. They also observed that some crystals, having apparently no screw dislocations at all emerging at the facet under study, grew in a very different manner, probably by quantum nucleation of terraces beyond a critical overpressure of several mbars. In this article, we show that we have found not much difference between crystals grown as slowly as Ruutu et al. and our previous crystals, as if growth from superfluid liquid $^4$He always led to a dislocation density in the range of 10$^4$–10$^6$ cm$^{-2}$. After a detailed description of our experimental methods and an analysis of our data, we shall discuss the possible origin of the differences between our dislocation density measurements and those of Ruutu et al. and add some comments about the nucleation of dislocations during growth.

## Experimental Methods

Most crystals have been grown from natural helium whose isotopic purity was found to be $25$ ppb, while it is usually assumed to be in the range of $0.1$–$0.3$ ppm. As explained below, we have also used ultrapure $^4$He with $0.4$ ppb and $5$ ppt $^3$He concentrations in order to see if the isotopic purity has any effect on the crystal’s quality. This is an opportunity to say that, contrary to wide spread belief, the isotopic purity of “natural helium” is very far from being universal. As shown by Oxburgh et al. [15] the $^3$He concentration in helium extracted from gas wells may vary by $2$ orders of magnitude from one site to another. Most of the natural $^4$He used to come from Texas where the $^3$He concentration is rather high ($0.1$–$0.3$ ppm) compared to other regions like the North Sea, Algeria or Qatar where it may reach a radiogenic limit that is $2$ % of the atmospheric concentration $R_a = 1.4$ ppm. Thanks to P. Jean-Baptiste^1^ and V. Dauvois,^2^ we obtained a measurement of the $^3$He concentration in the “natural” helium we had bought from the company Air Liquide: it is $25 \pm 1$ ppb, a low value that is consistent with its probable origin (Qatar).

### Design of the Sample Cell

**Fig. 1:**
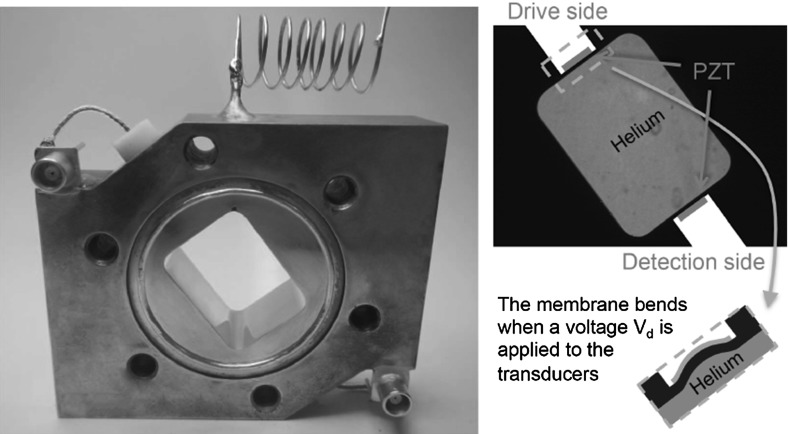
The acoustic cell without its windows. Its inner surface is polished and gold plated in order to avoid pinning of the liquid–solid surface during growth. During the growth, gravity forces the crystal to occupy the lower part of the cell. The coiled fill line emerges at the highest point of the cavity to avoid trapping liquid at the end of the crystal growth. Two piezoelectric transducers (PZT, diameter $5$ mm, thickness $0.25$ mm) glued on built-in copper membranes are used to drive and detect the acoustic resonances of the cavity filled with helium crystals (Color figure online)

**Fig. 2:**
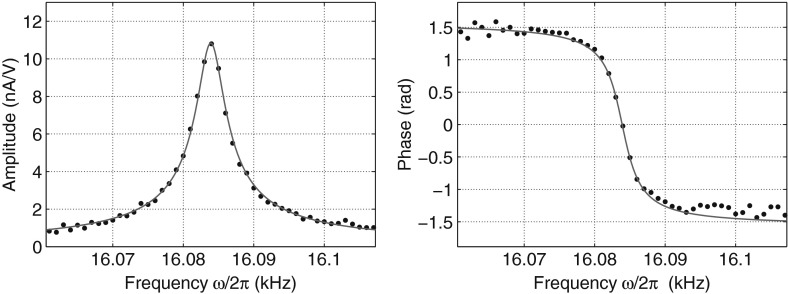
A typical resonance observed with the crystal P3 at $25$ mK, with a driving amplitude of $20$ mV. The dots correspond to the amplitude and the phase of the output current normalized by the drive voltage. The *red line* is a fit of the resonance using Eq. 1. The fitting parameters are $\omega _0/2\pi = 16084$ Hz, $Q=4124$ and $A=2.62$ pA/V (Color figure online)

In order to grow high quality $^4$He crystals, one needs to avoid stresses and to crystallize by injecting mass at constant temperature $T$ and pressure $P$, which is on a fixed point of the liquid–solid equilibrium line in the phase diagram. As explained by Pantalei et al. [12], to fill a cell with solid helium by crystallizing superfluid helium at low temperature is like filling a small volume with a non-wetting fluid because all solid walls—except properly oriented graphite—are preferentially wet by liquid helium [13, 14]. The solid–liquid interface does not enter corners, slits, nor cavities except if an overpressure is applied to overcome capillary effects. It also gets very easily pinned by any little defect or dust particle attached to walls. Then, in order to fill the whole cell by moving the liquid–solid interface upwards, one needs to use a fully convex cell with clean polished walls. We could not insert piezoelectric transducers inside the cell as done in previous experiments [3–7], in order to avoid any pinning of the liquid–solid interface during growth. It was necessary to study the stiffness of these crystals with an acoustic resonance method using transducers outside the cell. As indicated on Fig. 1, two piezoelectric transducers (diameter $5$ mm, thickness $0.25$ mm) are glued at the back of a thin part of the cell wall. This thin part is $1.85$ mm thick. It acts as a membrane that is bent under pressure. The transducer measures its deformation, which is in turn sensitive to the local acoustic pressure inside the cell. We have excited acoustic resonances in the cell with one transducer and detected them with the other transducer as shown by the recordings on Fig. 2.

Figure 1 shows the cell that was used for the present study. It is a $15\times 20$ mm hole with rounded corners made in a copper plate that is $15$ mm thick and attached to the mixing chamber of a dilution refrigerator. The coordinate system is such that the $y$ and the $z$ axis are along the shortest and longest dimension of the hole of the cavity, while the $x$ axis is along the thickness. The orifice of the fill line emerges at the highest point of the cell, otherwise it is blocked before the cell is fully crystallized. The cell walls were polished and gold plated before being closed by sapphire windows which allow optical control of the growth and a determination of the crystal orientation from the facetted shape of crystal seeds.

### Crystal Growth

In an attempt to grow crystals with the lowest possible dislocations densities, we have grown crystals at low temperature and small vertical growth speed. The growth surface is rough and consequently continuous, in contrast to facetted growth studied by Ruutu et al., which requires screw dislocations or nucleation of atomic planes.

The crystal seeds are produced by increasing the liquid pressure in the cell and reaching melting pressure. A crystal nucleates somewhere on the wall and drops down to the bottom as soon as its size is larger than the capillary length $l_c \approx$ 1 mm. This is because the temperature in the cell is highly homogeneous so that gravity is relevant. Being denser than the liquid, the crystal stays at the bottom of the cell. For every crystal seed, a fast and facetted growth ($>\!\!1$ mm/s) is used to determine the orientation of its sixfold axis of symmetry (c-axis). It is deduced from the position of the crystal edges, which are visible on the pictures captured during the fast growth. Table 1 shows such pictures, together with the orientation of the c-axis. After the orientation is determined, the crystal seed is reduced below its original size, typically $1$ mm (as done by Ruutu et al. [10]), and regrown to fill the whole cell.

**Table 1:** Compilation of results obtained with different crystals, with the c-axis of each crystal indicated by a red arrow (Color figure online)

		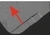	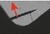	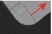	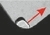	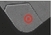	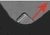	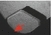
		P0	P1	P2	P4	P6	R2	T2
$x_3$	($\times 10^{-9}$)	25	25	25	25	25	0.4	0.005
$T_g$	(K)	1.39	1.40	1.40	0.029	0.020	0.018	0.018
$v_g$	($\upmu$m/s)	10	34	16	0.41	0.27	0.41	0.41
$\Delta c_{44} /c_{44}$	(%)	57	59	61	90	90	79	80
$Q^{-1}/\omega T^3$	($\times 10^{-5} s/K^3$)	2.3	1.5	0.22	3.3	3.7	2.5	2.25
$L$	($\upmu$m)	211	168	63	201	213	186	176
$\Lambda$	($\times 10^{5}$ cm$^{-2}$)	1.5	2.7	20.5	11.6	10.4	5.6	6.7
$\Lambda L^2$		69	75	82	471	471	197	209

The properties of the dislocation network have been studied as a function of the orientation, the growth speed $v_g$, the growth temperature $T_g$, and the $^3$He concentration $x_3$. For comparison P0, P1, and P2 were grown at high temperature and growth speed. They are expected to have a lower quality in regards to dislocation density and length. On the contrary P4, P6, R2, and T2 are different attempts to produce high quality crystals

The crystals selected for study had c-axes which were not vertical, and therefore the slow growth was continuous from a horizontal rough interface. The vertical growth speed of the crystal is adjusted by controlling two mass flow controllers installed on our gas handling system, and it can be varied from few tenth of nm/s up to about $100$ $\upmu$m/s. Our attempts to produce dislocation free crystals were performed at growth speeds of $270$ to $410$ nm/s, for which Ruutu et al. observed a particular growth mechanism without screw dislocations. For the typical growth speed $270$ nm/s used in this study, it takes one day to fill the entire cell. In parallel, a camera captures frames at regular intervals to monitor the growth.

### Resonance Method

The acoustic resonance method requires one to deduce the mechanical properties of solid helium from the resonance spectrum of the cavity filled with the oriented crystal. More precisely we had to measure the values of the elastic coefficient $c_{44}$ and the dissipation $Q^{-1}$ associated with the motion of dislocations.

First of all, in order to separate the signal coming from the resonance of the cavity from other signals (capacitive cross-talk between transducers, resonances in the copper cell), a preliminary spectrum was acquired with an empty cell. This background spectrum was subsequently subtracted from every acquired spectrum to keep only the signal $S(\omega )$ coming from the resonant cavity. Then, each resonance spectrum $S(\omega )$ was normalized by the drive voltage $V_d$ and fitted with the response function of a damped driven harmonic oscillator:

1 $$\begin{aligned} \frac{S(\omega )}{V_d} = \frac{A}{1-(\omega /\omega _0)^2+i\,Q^{-1}\omega /\omega _0} \end{aligned}$$

The strength of the resonance $A$ depends on the piezoelectric coefficients of the transducers and their coupling to the sample. It is left as a fitting parameter, as well as the quality factor $Q$ and the center frequency $\omega _0$. $Q$ and $\omega _0$ are used to deduce the dissipation in the crystal and the value of the elastic coefficient $c_{44}$.

Figure 2 shows a resonance example recorded at $T=25$ mK, a temperature well below the stiffening temperature at which $^3$He atoms start to pin the dislocation lines. It implies that dislocations are fully pinned by $^3$He impurities. $Q$ is consequently high ($4124$) since it is only limited by the acoustic coupling between the crystal and the body of the cell. Most of the time, the dissipation associated with dislocation motion is much larger than losses due to acoustic coupling. It is then reasonable to take $Q^{-1}$ as a value for the dissipation.

**Fig. 3:**
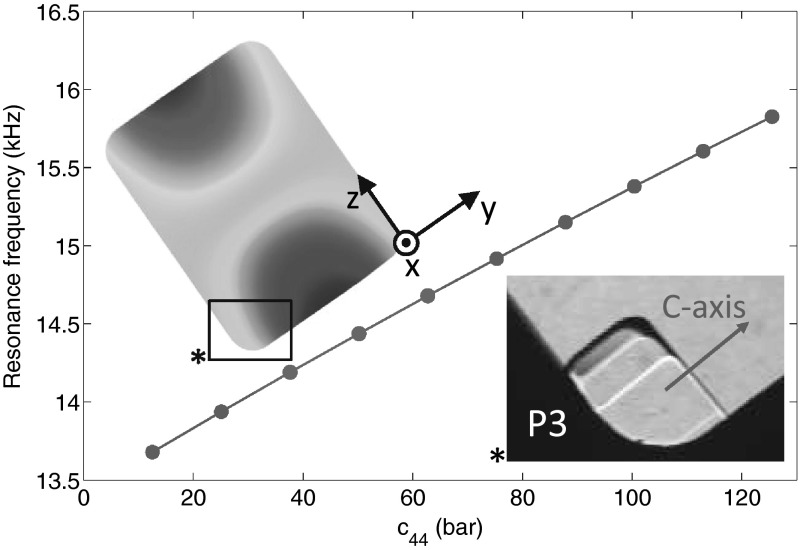
The resonance frequency of P3 as a function of $c_{44}$ computed with a numerical model of the cavity. A picture of the crystal is shown on the right *inset* with its sixfold symmetry axis along the $y$ axis. The left *inset* shows an effective pressure field of the vibration mode studied for P3, defined as the trace of the stress tensor. The location of the pressure anti-nodes makes this vibration mode strongly coupled to our detection system (Color figure online)

For every oriented crystal, a numerical model of the resonant cavity was produced with COMSOL software and used to predict the dependence of the resonance frequency on the value of $c_{44}$. An example for the crystal P3 is shown on Fig. 3, for which the sixfold axis of symmetry is almost along to the $y$ axis. For this crystal, when $c_{44}$ is varied from zero to its intrinsic value $c_{44}^{el}$, the resonance frequency varies almost linearly from $13.5$ to $16$ kHz. This calibration curve is subsequently used to convert the measured resonance frequencies into $c_{44}$ values. The accuracy of the numerical model was tested with several crystals in a fully pinned regime, where the value of $c_{44}$ is equal to the intrinsic value measured by Greywall [16] at $1.2$ K and $10$ MHz. Among different crystal orientations, the disagreement between the predicted value and the measured value of the resonance frequency never exceeded $2$ %. For example, for the measurement presented in Fig. 2, the measured frequency is $16.084$ kHz, while the predicted value is $15.734$ kHz. These $2$ % are likely due to a slight error in the cell size measurement and, in any case, compatible with Greywall’s error bars. This agreement confirms the validity of our numerical calculation.

**Fig. 4:**
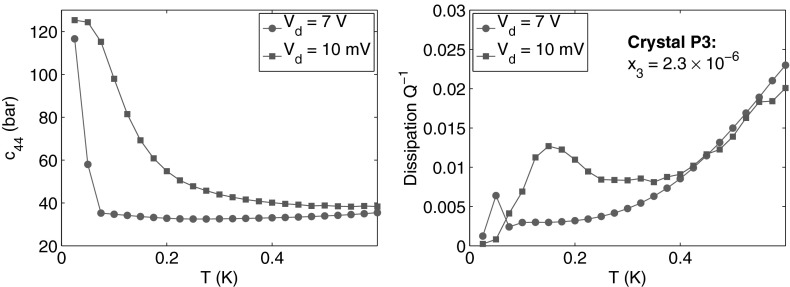
When a large drive voltage is applied (*red circles*), the high strain prevents $^3$He atoms from binding to the dislocation lines. As a result, the stiffening transition is shifted to lower temperature. In absence of $^3$He binding, the only damping mechanism comes from the interaction with the phonons as expressed in Eq. 3. From the high drive measurement and Eqs. 2 and 3, one can deduce the mean network length $L_N$ and the dislocation density $\Lambda$ (Color figure online)

One difficulty with this resonance method is that the crystal stiffness is not measured at constant strain amplitude but at constant driving amplitude. With quality factors as large as 4124 when the dislocations are fully pinned by $^3$He atoms, the strain magnitude can be much larger at resonance than out of it. In order to avoid large strains in the helium, which could detach $^3$He impurities from dislocations or create disorder, we have used very small driving amplitudes, typically $20$ mV meaning maximum strains $\epsilon \sim 10^{-7}$ at the acoustic resonance for $Q=4124$. Figure 4 shows that applying a larger drive amplitude shifts the softening transition down in temperature by a large amount.

### Dislocation Density and Network Length

To determine the dislocation density $\Lambda$ and the mean network length $L_N$, we have used the same method as in our previous work [5]. The relative softening $\Delta c_{44}/c_{44}^{el}$ and the dissipation $Q^{-1}$ associated with the dislocation motion are measured on cooling the crystal with a large drive voltage. In this regime, it is observed that the dissipation does not depend on $^3$He concentration $x_3$ nor on the driving amplitude $V_d$ [5], and phonon damping is the only dissipation mechanism. At low temperature, when the phonon damping is small, the relative softening can be expressed as

2 $$\begin{aligned} \frac{\Delta c_{44}}{c_{44}^{el}}= \frac{c_{44} - c_{44}^{el}}{ c_{44}^{el}} = \frac{\alpha \Lambda L_N^{2}}{1+\alpha \Lambda L_N^{2}} \mathrm ,\quad with \quad \alpha = \frac{32 (1-\nu )}{\pi ^4\ln (R/r)} \end{aligned}$$

and the magnitude of the dissipation:

3 $$\begin{aligned} Q^{-1} =\frac{\Delta c_{44}}{c_{44}^{el}} \omega \tau _{ph} = \frac{\Delta c_{44}}{c_{44}^{el}} \omega \frac{B_{ph} L_N^{2}}{\pi ^{2} C} \mathrm ,\quad with \quad B_{ph} = \frac{14.4 {k_B}^3}{{\pi }^2 {\hbar ^2}{c^3}} T^3 \end{aligned}$$

In Eq. 2, the constant $\alpha$ is expressed as a function of the Poisson’s ratio $\nu =0.3$, the typical dislocation spacing $R\simeq 100$ $\mu$m and the diameter of the dislocation core $r\simeq 1$ nm, so that $\alpha =0.019$. In Eq. 3, the quantity $C=\mu _{el} b^{2}\ln (R/r)/[4 \pi (1-\nu )]$ denotes the effective tension of the dislocation line, with the dislocation’s Burgers vector $b=3.7\times 10^{-10}$ m. A good approximation is $C = 2.3\times 10^{-12}$ N. In the phonon regime, the dislocation damping $B_{ph}$ is dominated by the fluttering mechanism as shown in [5]. It is expressed as a function of the Debye sound speed $c=256$ m/s [17]. From Eqs. 2, 3 and the measurement of $\Delta c_{44}/c_{44}^{el}$ and $Q^{-1}$ in the phonon regime, one can easily derive the value of $\Lambda$ and $L_N$.

## Experimental Results

### Search for Dislocation Free Crystals

Table 1 shows results obtained for various crystals. Crystals P0 to P6 were grown from natural helium, while crystal R2 was grown from our ultra pure “ppb helium” that contains $0.4$ ppb of $^3$He and crystal T2 from our “ppt helium” that contains only $5 \pm 1\times 10^{-12}$ of $^3$He. The latter extreme purity helium was provided to us by P. McClintock (Lancaster University) and its purity measured by Ken Farley (Caltech, USA).

For experimental comparison to crystals grown at low temperature and slow growth speed, P0, P1, and P2 were grown in a similar fashion to Haziot et al. This growth method may result in a higher dislocation density. They were grown at $T_g=1.4$ K at relatively large velocities $v_g$: $10$–$34$ $\upmu$m/s and then cooled down to $1$ K. This is to ensure that the whole cell was crystallized, even in thin slits between windows and the copper plate. Indeed, when cooling down from $1.4$ K to $1$ K, the melting pressure $P_{\mathrm{eq}}$ decreases by about $1$ bar. This is enough to force the solid–liquid interface to enter these slits, but may produce stresses larger than the yield stress of helium crystals. Although the high density of thermally activated vacancies above $1$ K may help maintain a good crystal quality during this procedure, this is obviously not the best way to achieve very high crystalline quality.

The values of $\Lambda$ and $L_N$ for crystals P0, P1, and P2 are similar to what had been already found by Haziot et al. [5]. It should be noticed that P2 has a higher dislocation density as well as a shorter network length. This might be the result of large stresses that occurred close to the end of this particular growth, when trying to melt a plug that formed in the fill line.

In order to improve the crystal quality, crystals P4 and P6 were grown under growth speeds and temperatures similar to those used by Ruutu et al. : $v_g=410$ nm/s at $T_g=29$ mK for P4 and $v_g=270$ nm/s at $T_g=20$ mK for P6. Under these conditions the crystals stay in equilibrium with thin liquid regions underneath windows. These liquid regions may trap $^3$He impurities. They force the cell pressure to follow the melting line but the corresponding variations are less than $5$ mbar as long as the temperature is kept below $650$ mK as we did. These two crystals showed large network lengths and record values of the softening $\Delta c_{44} /c_{44}=90$ %, consequently record values also for the dimensionless quantity $\Lambda L_N^2$. It means a very large degree of dislocation alignment in sub-boundaries. However, crystals P4 and P6 have dislocation densities similar to the previous ones, of order $10^6$ cm$^{-2}$. In summary, the quality of crystals grown slowly at low temperature is not significantly different from those grown at higher T or at higher speed.

Since one may relate the dislocation nucleation to the presence of impurities, we then used very high purity helium. Crystals R2 and T2 do not show a significant improvement in terms of dislocation density or network length as compared to P0, P1, and P2. In summary, it appears that, when grown from superfluid helium, crystals always present dislocation densities in the range of $10^4$–$10^6$ cm$^{-2}$ and dislocation lengths from a few ten to a few hundred micrometers. These results are in contrast to those obtained by Ruutu et al. [10, 11]. We discuss it below, in the last section of this article.

### Behavior of Extreme Purity Crystals

The study of extreme purity crystals such as T2 was an opportunity to study the effect of annealing on the properties of crystals. Rojas et al. had noticed the particular properties of crystals freshly grown at low temperature [19]. Their natural purity crystals were kept in contact with some liquid that acted as a trap for $^3$He impurities. At low temperature, this trapping was so efficient that the $^3$He concentration in the bulk solid was significantly modified. As a result, the dislocations were not fully pinned and the crystal stiffness was reduced. When warmed up for the first time, these freshly grown crystals showed some stiffening around $0.1$ K instead of the usual softening. Rojas et al. proposed that this stiffening was due to some $^3$He impurities escaping from the liquid trap and binding to dislocations. But this effect was possibly mixed with some annealing occurring in the dislocation network.

**Fig. 5:**
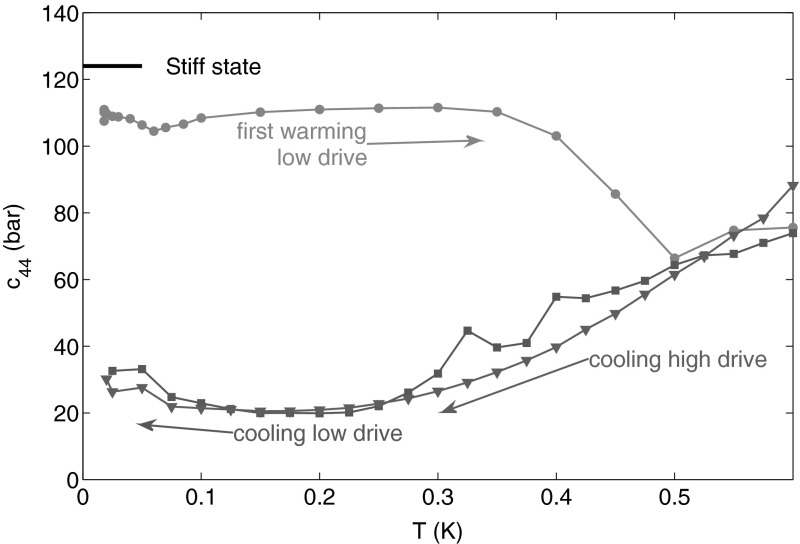
Crystal T2 with a negligible $^3$He concentration of $5\times 10^{-12}$ is an opportunity to study annealing in helium crystals. Only during the first warming (*blue circles*), around $0.4$ K, a large softening occurs probably due to annealing of jogs. After this annealing, this exceptional crystal remains soft even at low temperature and low drive voltage (*green squares*) because there are no $^3$He atoms to bind to dislocation lines (Color figure online)

This crystal T2 represents an unusual situation where the $^3$He concentration was negligible, regardless of the temperature. We could look for annealing effects in the absence of impurity binding effects and demonstrate the remarkable mobility of dislocations. Figure 5 shows that fresh from growth, the value of $c_{44}$ for T2 is close to the intrinsic value, only depleted by a small amount. No significant changes happen up to $0.5$ K, at which a large softening occurs. After this noticeable event, the dislocation network reaches a stable configuration and successive temperature cycles show a reproducible softening of $80$ % at low temperature. If the dislocation density is unchanged, this means that the pinning length has increased to a large value (here 200 $\mu$m) during the softening at $0.5$ K. It suggests that, in freshly grown crystals, the network length is initially reduced by the presence of jogs. These jogs are annealed out at $0.5$ K thanks to the thermal activation of vacancies, after which the network length is only limited by the network nodes. Annealing effects around $0.5$ K have been observed in other experiments, for example by Suhel and Beamish [20], when studying the relaxation of pressure gradients.

## Discussion

Compared to crystals that were grown more rapidly ($10$–$34$ $\upmu$m/s) at higher temperature ($1.4$ K), such as P0, P1, and P2, we have shown that the dislocation network length may be larger in crystals grown slowly such as P4 and P6 (a few hundred nm/s) at low temperature (below 30 mK). However, we have not found a lower dislocation density in P4 and P6, and the latter is larger by several orders of magnitude than the screw dislocation densities found by Ruutu et al. [10, 11]. Compared to crystals grown in previous cells whose shape was much more complicated with much rougher walls, the present cell has not allowed us to significantly improve the quality of our crystals. Apparently, crystals grown from superfluid liquid helium at constant T and P have dislocation densities always in the range of 10$^4$–10$^6$ cm$^{-2}$. These results call for several remarks.

Kosevich and Svatko [18] had proposed that one mechanism of dislocation formation was local stresses associated with the wall roughness. However, we have found dislocation densities that are similar in the present cell with polished walls and in previous experiments [4–7] where crystals were grown between two piezoelectric transducers that had rather rough surfaces.

During growth, it happened from time to time that the solid–liquid interface was pinned by a local dust particle on windows. This pinning depends on the presence of facets especially if they touch the window, consequently on crystal orientation and growth speed (a high speed favors the presence of large facets). Usually, the interface unpins when the height difference between the dust particle and the flat part of the interface far from the dust particle exceeds a few millimeters. When it unpins, the interface jumps up, which could create dislocations. However, the stresses involved should not exceed few millimeters of hydrostatic pressure difference. That value is small compared to the yield stress of helium crystals that is larger than a few millibars [20, 21], and is unlikely to create plastic deformations. Consequently, pinning and the associated jumps should not create dislocations, which could have explained why our dislocation densities are so much larger than Ruutu’s.

One may think that the final dislocation density is a simple consequence of the number of dislocations in the seed from which the whole crystal is grown. This is why we have melted our crystals to a small size before regrowing them slowly. To control the melting below a $1$ mm size is practically impossible because pressure fluctuations easily melt small seeds. Such pressure fluctuations can be induced, for example, by temperature or level fluctuations in the $4$ K bath of the refrigerator. Ruutu et al. [11] have grown crystals from seeds with a size similar to ours.

In our opinion, the main reason why our dislocation densities are so different from those found by Ruutu et al. must be that, in reality, we are not measuring the same quantity. Ruutu measured a minimum distance between screw dislocations of opposite sign emerging in a c-facet. We measure a softening that is related to a density of *mobile dislocations.* It is likely that these mobile dislocations are the edge dislocations forming the sub-boundaries imaged by Iwasa et al. [9], which glide parallel to the basal planes. Indeed these dislocations are split into partial dislocations separated by a stacking fault parallel to the basal planes and Legrand [22] has shown that this splitting is what determines the gliding direction in hcp crystals. One may argue that, in the sub-boundaries, the dislocation character is not purely edge nor purely screw as explained by Iwasa [9] and in agreement with Fefferman’s finding [7] of a distribution of $^3$He binding energy to these dislocations. But these sub-boundaries are planes perpendicular to the basal planes and the dislocations inside are parallel to these basal planes and their Burgers vector also, whether purely edge or partially screw. In contrast, what is measured by Ruutu et al. is a distance between screw dislocations intersecting c-facets, with a Burgers vector parallel to the c-axis. So, the screw dislocations that contribute to the growth of c-facets are not the same as the ones forming sub-boundaries. Furthermore, the mutual distance between the screw dislocations considered by Ruutu et al. cannot be simply related to a general dislocation density in the crystal because they are not arranged in an isotropic 3D network. In summary, the dislocations responsible for the growth of c-facets are not the same as the dislocations whose high mobility reduces the elastic coefficient $c_{44}$.

As for the nucleation mechanism which leads to a dislocation density in the range of 10$^4$–10$^6$ cm$^{-2}$ in all $^4$He crystals grown at constant *T* and *P* as we usually do, it does not seem to be related to the concentration of $^3$He impurities either, so that it remains basically unknown up to now.

## Footnotes

1 P. Jean-Baptiste, LSCE-IPSL, CEA Saclay, France, personal communication.

2 V. Dauvois, DEN, DANS, DPC, SECR, LRMO, CEA Saclay, France, personal communication.
